# Personality Disorder Diagnoses in ICD-11: Transforming Conceptualisations and Practice

**DOI:** 10.32872/cpe.9635

**Published:** 2022-12-15

**Authors:** Michaela A. Swales

**Affiliations:** 1 North Wales Clinical Psychology Programme, Bangor University, Bangor, Wales, United Kingdom; University of Zurich, Zurich, Switzerland

**Keywords:** personality disorder, severity of personality disorder, ICD-11, trait domains

## Abstract

**Background:**

Until the advent of the ICD-11, classification of personality disorders was based on categorical prototypes with a long history. These prototypes, whilst familiar, were not based in the science of personality. Prototypical classifications were also complex to administer in non-specialist settings requiring knowledge of many signs and symptoms.

**Method:**

This article introduces the new structure of ICD-11 for personality disorders, describing the different severity levels and trait domain specifiers. Case studies illustrate the main aspects of the classification.

**Results:**

The new ICD-11 system acknowledges the fundamentally dimensional nature of personality and its disturbances whilst requiring clinicians to make categorical decisions on the presence or absence of personality disorder and severity (mild, moderate or severe). The connection between normal personality functioning and personality disorder is established by identifying five trait domain specifiers to describe the pattern of a person’s personality disturbance (negative affectivity, detachment, dissociality, disinhibition, and anankastia) that connect to the *Big 5* personality traits established in the broader study of personality.

**Conclusions:**

Whilst new assessment measures have been and are in development, the success of the new system will rely on clinicians and researchers embracing the new system to conceptualise and describe personality disturbances and to utilise the classification in the investigation of treatment outcome.

## Highlights

- Introduces the new structure of ICD-11 for personality disorders.
- Describes the different severity levels and trait domain specifiers.
- Case studies illustrate the main aspects of the classification.
- Discusses the issue of stigmatization in clinical practise.

## Problems With ICD-10: The Case for Change

Personality disorder is perhaps the most stigmatising diagnosis to receive (Bonnington & Rose, 2014). We all have a personality and our personality is often central to how we perceive ourselves in the world. So, to be told that this part of ourselves – or indeed our whole self – is disordered is extremely stigmatising and potentially highly damaging. Thus, for a clinician to make the diagnosis they must be sure that the benefits outweigh the costs. There are now a number of treatments developed for people who experience the problems that commonly are labelled personality disorder, particularly borderline personality disorder (Storebø et al., 2020), and therefore the cost benefit ratio has changed. In this context, withholding the identification of problems for which there are effective interventions becomes a different ethical challenge, whether the diagnosis is stigmatising or not.

How clinicians conceptualise personality disorder impacts their ensuing discussions with their clients and patients about the diagnosis. These discussions provide significant opportunities to mitigate stigma, especially as evidence indicates that it is often mental health professionals who hold the most stigmatising views of all (Newton-Howes et al., 2008; Ring & Lawn, 2019). ICD-10 like the DSM, was based in clinically derived prototypes that were not based in scientific research that can, as Tyrer and Mulder (2022) argue, be traced back to the conceptualisations of Schneider. Each of the ten prototypes (personality disorders) had a substantial list of symptoms which meant that making a diagnosis required clinicians to be familiar with a long list of symptoms and how they related. Often these symptoms overlapped. Such complexity presented particular challenges in the many low and middle income countries using the classification where there are very few psychiatric specialists, much less personality disorder experts. This inherent structure of the classification resulted in two significant problems. Firstly, rarely did clinicians use anything other than three of the diagnostic categories (Emotionally Unstable Personality disorder; Antisocial Personality Disorder; and Personality Disorder Not Otherwise Specified), making the remainder of the classification effectively redundant and also raising questions about its utility. Secondly, often people met criteria for more than one, sometimes many more than one, personality disorder diagnosis resulting in multiple ‘comorbidities’ which were more apparent than real. Consequently, some individuals were loaded up with diagnoses providing added stigma with no realistic prospect of benefit. In response to these not insignificant problems, ICD-11 fundamentally changes the way in which personality disorder diagnoses are conceptualised. It recognises that personality and personality disorder are continuous with each other, and although a categorical structure is maintained, the system recognises that the underlying structure is dimensional. The new system also establishes a connection between basic personality research and the diagnosis of personality disorder.

In fundamentally changing the structure of personality diagnosis ICD-11 provides the potential for a more compassionate framing of personality disorder in discussions between clinicians and the people who come to them requiring help. To mitigate stigma clinicians must root their discussions of personality and its disorders in a psychological understanding of the development of personality rather than within the terminology of psychiatric nosology. Personality develops in the transaction between our biology and our early life experiences. Personality characteristics have a strongly heritable component (Vukasović & Bratko, 2015) and can be seen in early temperament, which has a high degree of stability across the life span (Roberts & DelVecchio, 2000). Early trauma, however, can have a significant impact on the developing brain. These impacts may make a child more sensitive, or aggressive further prompting adverse experiences such as invalidation or punishment from caregivers which may increasingly impact the child’s neurobiology. Thus, personality and personality disorder develop in the transaction between biology and environment and can be conceptualised as a person’s best efforts to function and cope with their familial and social environment given their biological heritage and early life experiences. Conceptualising personality dysfunction as learned patterns of coping – which may have been functional in the person’s early context, and may continue to function in some environments – that have become problematic for the person, potentially provides a supportive and less stigmatising context in which to discuss personality and its disorders. ICD-11’s new structure which is strongly connected to the study of human personality provides a context for furthering these initial discussions with clients and patients. A study with health professionals of the respective utility of ICD-10 versus ICD-11 found that the new structure was more useful with respect to formulating interventions, communicating with clients, comprehensively describing a person’s difficulties and ease of use (Hansen et al., 2019). Whether clients themselves experience clinicians’ discussions using the new structure as less stigmatising will require systematic research. If this aspiration is to be realised, initial service user responses indicate that clinicians will need to be more adept at understanding internal distress and that patterns of behaviour were adaptive responses to early adversity (Hackmann et al., 2019).

### Aims of the New Classification

Simplification and greater utility are the primary aims of the new classification. The initial two step-process of diagnosing PD (do the person’s difficulties meet the threshold for disorder and, if they do, how severe are they) are much simpler than the previous system and therefore potentially more clinically useful, especially in non-specialist settings. The new system removes the artificial comorbidity of ICD-10 and also significantly decreases the number of symptoms clinicians need to assess in determining the diagnosis thus potentially improving clinical utility. Focusing on severity explicitly foregrounds risk, potentially improving the identification of risk in clinical settings. Severity directly links to treatment intensity, frequency, setting and level of care required, thus, helping services to decide on the complexity of interventions required (Bach & Simonsen, 2021). Whether the classification delivers on these aims will be a matter for subsequent research and implementation studies to decide. What follows is a description of the changes in ICD-11, illustrated by three case studies, and a discussion of issues in assessment.

## Description of the Changes

In sum, the new diagnostic classification requires two steps with two further optional steps if required. In the first step clinicians assess whether the person’s difficulties meet the general requirements for a personality disorder diagnosis. Secondly, if these requirements are met, then clinicians further assess to determine the severity of the difficulties. The third and first optional step requires further assessment of the person’s personality trait domains to more comprehensively describe an individual’s personality disturbance. Finally, and if applicable, a borderline pattern specifier can be applied. Each of these steps will be considered in further detail.

### Description of the Core Features of Personality Disorder

The central features of personality disturbance in ICD-11, as in DSM-5, are disturbances in aspects of both self and interpersonal functioning. For a diagnosis, these disturbances must be enduring – so present for a minimum of two years. Self-dysfunction may manifest as persistent difficulties in maintaining a stable sense of identity, a pervasive sense of impoverished or highly over-valued self-worth, inaccuracies in self-perception or challenges in self-direction and decision making. Persistent difficulties in making and sustaining close relationships or in the ability to understand other people’s perspectives are typical manifestations of the interpersonal dysfunction. Managing conflict in relationships may also present significant challenges. These two main features will manifest in maladaptive patterns of cognition, emotional experience and expression and behaviour which must be evident across a range or personal and social situations.

When considering the disturbance demonstrated or described by the person there are several important factors to consider. First, the disturbance must be present across a range of personal and social situations and not limited to single contexts, although, particular types of situation or common prompting events may elicit the same behaviour across contexts. For example, a person may become repeatedly aggressive when their views are contradicted and this pattern maybe evident with family, and in both social and work contexts. Secondly, when working with young people the developmental context must be considered. Interpersonal difficulties and a degree of unstable self-identity are developmentally normative during the adolescent period. Clinicians, therefore, must be certain that the behaviours reported or demonstrated are significantly different to behaviour of young people of that age and developmental stage within their specific cultural context. Clinicians must carefully assess whether the young person’s behaviours are normative responses to adverse environmental situations. For example, a young person may run away from home frequently, getting into fights, using drugs and self-harming because they are being physically and sexually abused at home. Similar difficulties may arise in the situation of women subjected to coercive control and domestic violence and in both cases the person may have significant difficulties in alerting the assessor to the truth of the situation they find themselves in. A proper assessment of context, therefore, is required to ensure that presenting problems truly warrant a diagnosis of personality disorder. Third, and following on from the previous point, the disturbance must not be explained primarily by social and cultural factors, including socio-political conflict. Assessors must take especial care when assessing a person from a different culture or heritage to their own to guard against their own culturally defined assumptions about behaviour, thought and emotional expression. Fourth, the disturbance must not be a direct effect of medication or of some other substance, including withdrawal effects. Finally, the disturbance must be associated with substantial distress of significant impairment in personal, family, social, educational, occupational or other important roles.

### Severity Ratings

Once a determination has been made that a person’s disturbance meets threshold for a personality disorder diagnosis, the severity of that disturbance (mild, moderate or severe^1^1Sub-threshold difficulties which present problems in specific contexts (e.g. in effectively accessing healthcare) may be coded as *Personality Difficulty*, which can be found in the section of the ICD-11 classification *Factors Influencing Health Status or Contacts with Health Services*.) needs to be considered. Researchers recently have argued for the importance of severity from a conceptual and methodological perspective (Pincus et al., 2020; Sharp & Wall, 2021). Selecting this feature as the next required feature of diagnosis, however, relates to the strong relationship between severity and clinical outcomes (Clark et al., 2018; Crawford et al., 2011; Yang et al., 2010). Severity is determined by several factors:

- The degree and pervasiveness of disturbance in the person’s relationships and their sense of self
- The intensity and breadth of the emotional, cognitive and behavioural manifestations of the person’s disturbance
- The extent to which these patterns and problems cause distress or psychosocial impairment
- The level of risk of harm to self and others.

As personality disorder becomes more severe an increasing number of areas of a person’s life become affected by their difficulties and evidence of harm to self or others becomes more prevalent. For example, in mild personality disorder a smaller number of areas of a person’s life will be affected, for example, work and close friendships but perhaps not family or hobbies; or if the difficulties affect all of these areas, they will be mild in severity. Severe personality disorder in contrast affects all areas of a person’s life, will be clearly evident to other people around them and will always entail harm to self or others.

#### Mild Personality Disorder

The most notable aspect of mild personality disorder is that only some areas of personality function are affected. For example, a person might have difficulty making decisions or deciding on the direction of their career yet have a strong sense of self-worth and identity. Problems in many interpersonal relationships or in the performance of social and occupational roles are evident but some relationships are maintained or social roles carried out. The manifestations of a person’s difficulties are generally mild and not typically associated with harm to the self or others. For example, they may struggle to recover from minor setbacks or criticisms when stressed or they may distort how they perceive situations or other people’s motives without losing total contact with reality. Whilst the personality disturbance may be mild, the person may still experience substantial distress and impairment. The distress and impairment are limited to a narrower range of functioning or, if the difficulties are across many areas, the difficulties are less intense.

Text Box 1 **Mr R: Mild Personality Disorder With Negative Affectivity and Anankastia**

Mr R is 54 years old and has been referred for assessment by his employer. He arrives at the appointment with his sister with whom he has lived for 15 years since the breakdown of his marriage.

Mr R describes how he was recently promoted to head up a team to run a major project. He was promoted because of his track record of delivering high quality work on time. For the first time he has been required to both lead and co-ordinate a team. His high standards and desires for perfection have caused difficulties with colleagues infuriated by Mr R’s exacting standards and frequent requests for work to be re-done. Previously when working alone co-workers have tolerated his style of working because it had minimal impact on them.

Mr R was previously married and has three children. He describes his former wife as exceptionally difficult to live with as she was ‘extremely untidy, disorganised and slovenly’. They disagreed about how to raise their children and he found his children’s ‘noise and chaos’ impossible. He laments that children are no longer ‘seen and not heard’. In a separate interview with his sister, she reports that Mr R is extremely punctilious about household standards and she thinks that his wife was no untidier and more disorganised than most people. They live effectively together by having separate spaces in their old family home so that she is not impacted by his standards – except in the kitchen where she does not mind following his ‘rules’ about how things must be maintained. Mr R now sees his children, now adults, relatively often. He says he is surprised how well they turned out given their ‘chaotic start’.

Mr R is the secretary for his local cricket club and the local church. His organisational skills are much appreciated, although, he occasionally argues with other members of these groups when they disagree about how things should be organised.

Mr R (see Text Box 1) illustrates these features of mild personality disorder. Mr R has sustained his work history for many years and indeed his personality traits, of which more later, have served him well. Difficulties in the work context have only recently begun as a result of a change of demand necessitating more team working where his high standards have interfered with effective working relationships. His difficulties in close interpersonal relationships have been evident for many years within the family context, yet he is able to still maintain some social relationships and family connections.

#### Moderate Personality Disorder

For moderate personality disorder, disturbance affects multiple areas of personality functioning such as identity, sense of self, formation and maintenance of intimate relationships, capacity to control and moderate behaviour. Despite these difficulties, some areas of functioning may be relatively less affected. Occasionally moderate personality disorder will be associated with harm to self or others. When this is present, typically, it will be of moderate severity.

Text Box 2 **Ms T: Moderate Personality Disorder With Negative Affectivity and Disinhibition (Borderline Pattern Specifier)**

Ms T is a veterinary student, aged 26. Her course tutor suggested that she seek assistance as her behaviour on her current programme of study is likely to lead to suspension of her studies if it does not change. This is not the first time that Ms T has presented to services. She describes a history of suicidal thoughts and self-harm behaviours that began in her middle teenage years. Whilst in her early twenties suicidal and self-harm behaviours were less common, they have increased in frequency following a series of break-ups of romantic relationships. Ms T describes that she often feels that she can no longer cope with her life and her emotions and that considering suicide and self-harm provides a degree of relief from the intensity of these thoughts and feelings. Ms T says that she believes she experiences emotions more intensely than other people.

Ms T describes intense and frequent mood changes that have worsened as a result of the interpersonal difficulties she has been experiencing. She describes intense emotions often in response to minor things. For example, her current presentation was prompted after she had yelled and thrown things during a meeting with her Programme Director and her other course mates where her next placement was being discussed and she had not got the placement that she had hoped for. She realised almost immediately that she had acted inappropriately and was extremely tearful and apologetic. Incidents like these have resulted in her peers treading carefully around her or avoiding her altogether. She discovered recently that she had not been invited on an outing and she believes this is a consequence of her reactivity.

Ms T describes a history of frequent romantic relationships. She falls in love rapidly and intensely. Recent relationships have ended as a result of the intensity of her attraction, her jealous rages and, when she believes her partner is unfaithful, she herself then initiates casual sexual contacts with other people.

Ms T’s parents were highly critical of her as she was growing up. Academic achievement was extremely important to them. She was very close to her grandmother and spent much of her early teenage years living with her as her parents travelled extensively with their work. Her grandmother suffered from a chronic illness and Ms T cared for her during this time and was devastated when she died when Ms T was 16. She describes her grandmother as the only supportive person in her life. After her grandmother’s death she would often run away from home for days at a time drinking heavily and initiating casual sexual encounters. Despite this she maintained good grades at school as she wanted to be a vet – an ambition her grandmother also had but was unable to fulfil.

Marked problems in interpersonal relationships will be evident. Relationships may be tumultuous, characterised by high levels of conflict and frequent ruptures. Alternatively, a person may be conflict avoidant and withdraw from relationships or they may be highly dependent on one or two relationships being either submissive or dominant.

Ms T (see Text Box 2) fulfils the requirements for moderate personality disorder as a much greater number of areas of functioning are affected. There is also evidence of harm to self. Her academic skill is well preserved, however, capitalising on her abilities in her chosen profession is compromised by her emotional regulation difficulties and their interpersonal consequences. Her social relationships are also heavily impacted.

#### Severe Personality Disorder

People with severe personality disorder have major disturbances in their sense of self functioning. For example, they may have no sense of who they are, experience intense numbness or report that what they believe and think changes dramatically from one context to another. Some individuals may have a very rigid view of themselves and the world and have very regimented routines and approaches to situations. A person’s sense of self may be grandiose or highly eccentric or characterized by disgust and self-contempt.

Unsurprisingly, virtually all relationships in all contexts are adversely affected. Often relationships are very one-sided, unstable or highly conflictual. There may even be a degree of physical violence. Family relationships are likely to be severely limited or highly conflictual. The person’s ability, and sometimes willingness, to fulfil social and occupational roles is severely impaired. So, for example, a person may be unwilling or unable to sustain regular work as a result of lack of interest, or effort, or poor performance. Alternatively, the poor work performance may derive from interpersonal difficulties or inappropriate behaviour such as angry outbursts or insubordination. Severe personality disorder is often associated with harm to the person or other people. Severe impairment is evident in all areas of the person’s life.

Text Box 3 **Mr D: Severe Personality Disorder With Detachment and Dissociality**

Mr D aged 34 has been referred for evaluation pending trial. He has been arrested on charges of befriending and then defrauding elderly people. Over the last ten years he has befriended 5 different elderly people, all of whom lacked family nearby. He would begin the relationship by introducing himself as a representative of a local charity that supported elderly people in organising practical tasks about their home e.g arranging gardeners, decorators etc. He would then spend increasing amounts of time with his intended victim and then pour out a story about how his mother had a serious medical illness for which treatment was only available in the US and how distressed he was that he could not afford it. He would eventually accept funds from his victims after protesting for a short while that he could not possibly accept their generosity. His victim’s reported that his persistent refusal over a period of time was in part what was so convincing. Mr D is confident that he will be found not guilty as he maintains that all of the money was given as ‘gifts’. He maintains that his victims were simply grateful to him for all the support and help that he offered them. His victims, in contrast, describe how he was initially helpful but latterly would easily become irritated and aggressive if they did not follow his advice and they found it hard to resist his suggestions.

Mr D in recent years has had no regular employment and has relied on the funds that he obtained from his victims to sustain himself. His family have severed all contact with him– including his mother- because of his constant demands for money and his aggressive behaviour when his demands are not met. He has no reliable place to live, frequently being asked to leave where he is living because of non-payment of rent. Mr D describes other people as a nuisance and as parasites and says that he can see no need of relationships or connections with others.

Mr D had difficulties originating in childhood. He described his father as an abusive man who frequently told him to stand up for himself. He often fought with other children and complained that he was constantly disrespected although he was often described as a bully. He left school with minimal qualifications and although he began a college course he was dismissed for a combination of non-completion of the course and aggressive behaviour towards other students.

Mr D (Text Box 3) presents with severe personality disorder. All areas of his life are affected. He has no meaningful relationships with family or friends and the only connections he has made are with his victims who he has exploited for personal gain. Yet he seems unwilling or unable to appreciate the damage and harm that he has inflicted upon them.

### Trait Domain Specifiers

Once the two obligatory steps for diagnosing PD are completed, there are two further optional steps both of which involve further describing the type of difficulties that a person presents with. In some jurisdictions the first two steps will be all that is required. In countries with more advanced systems in place for supporting people who receive a personality disorder diagnosis the first of these next two steps would be encouraged. As is evident from the descriptions of severity above, the manifestations of severity vary significantly, and these expressions are in accordance with the trait domains of normal personality function. ICD-11 describes five trait domain specifiers that are continuous with normal personality characteristics, consistent with the *Big 5* model of personality (McCrae & Costa, 1987) and have been found in most if not all mental disorders. Trait domain specifiers are not diagnostic categories rather they represent a set of dimensions corresponding to the underlying structure of personality in all people. Factor analytic studies broadly speaking support the ICD-11 five factor structure (Bach et al., 2017; Mulder et al., 2016), although some studies have found four factors rather than five, where one factor captures the two polar opposites of disinhibition versus anankastia (Bach et al., 2020; Oltmanns & Widiger, 2018). As many trait domain specifiers can be applied as are appropriate to describe a person’s characteristics. Individuals with more severe personality disturbance tend to have a greater number of prominent traits although it is possible to have severe personality disorder and manifest only one trait domain e.g. dissociality. Each of the trait domain specifiers will now be considered in turn.

#### Negative Affectivity

Tendency to experience a broad range of negative emotions forms the central element of negative affectivity. In people with a personality disorder diagnosis this typically means that they experience a broad range of negative emotions with a frequency and intensity that others judge as being out of proportion to the situation. Nevertheless, given the person’s life experiences and genetic heritage their responses make sense in terms of their own learned experiences. Common negative emotions include anxiety, worry, sadness, fear, anger, hostility, guilt and shame. The person often experiences emotional lability with accompanying difficulties in regulating their emotions. They are often easily distressed and it takes them longer than average for their emotions to return to their baseline levels.

As a result of intense and frequent emotions, negative thoughts and attitudes commonly occur which, in turn, further fuel strong emotional reactions. Hopeless thoughts are frequent and a tendency to assume that interventions or solutions suggested by friends, family and professionals will not help their situation. Individuals often have low self-esteem and self-confidence which may result in avoiding situations or activities as they anticipate difficulty. Often, they do find situations difficult, because of their emotional sensitivity. They may become highly dependent on others for advice, reassurance, help and direction. At times, they may be understandably envious of other’s abilities and successes given their own challenges. In more severe cases they may experience intense feelings of worthlessness and suicidal ideation.

Negative affectivity may be very evident both in a person’s report and behaviour, as might be seen in the case of Ms T or it may be heavily disguised and may not even be reported directly as is the case with Mr R. Interactions with other personality traits influence how negative affectivity manifests. In individuals with traits of greater disinhibition negative affectivity is more likely to be clearly evident and to present earlier in life, whereas in those with detachment and anankastia it may present later, be less directly evident and may even not be reported.

#### Detachment

Detachment can be either social or emotional. Social detachment in people with a personality disorder diagnosis consists of significant avoidance of social interactions and what they may consider unnecessary interpersonal contact. The person may often respond in ways that actively discourage social interaction. As a result, the person often lacks friends or even acquaintances, often avoiding intimacy of all kinds, including sexual intimacy. Emotional detachment is evident in a reserved and aloof manner with limited emotional expression and experience, both verbally and non-verbally. In extreme cases a person may report a lack of emotional experience altogether; they may be unreactive to positive or negative events and both report and demonstrate a limited capacity for enjoyment. Mr D shows evidence of both social and emotional detachment

#### Dissociality

Mr D also shows strong evidence of the dissociality trait specifier. Disregard for the feelings and rights of others which includes self-centeredness and lack of empathy is at the centre of this trait domain. People with this trait may demonstrate a sense of entitlement, expecting others to admire them. They may endeavour to attract the attention of others or to ensure that they are at the centre of other people’s attention. If others do not respond as they wish they may dramatically express their dissatisfaction. Dissociality may lead to a disregard of the importance of others and the person may have a relentless focus on their own needs, desires and comfort.

#### Disinhibition

Impulsive action in response to immediate internal or environmental stimuli without consideration of longer-term consequences forms the basis of the disinhibition trait domain. People with this trait tend to act rashly without considering the impact of their actions on themselves or others in the longer term and this can include putting themselves or others at risk. Difficulties delaying reward or satisfaction result in strong associations with such behaviours as substance use, gambling, and unplanned sexual activity. Alongside impulsive action, appraisal of risk is impaired combined with an absence of an appropriate sense of caution resulting in, for example, reckless driving, dangerous sports and activities without appropriate training and preparation. Ms. T shows elements of disinhibition in her reactions in romantic relationships and in her responses to her current placement.

People with this trait are frequently distractible, becoming easily bored or frustrated with routine, difficult or tedious tasks and may often be seen scanning the environment for more pleasurable options. People with a personality disorder with this trait often demonstrate a lack of planning preferring spontaneous over planned activities with a focus on immediate emotions and sensations with little attention to long-, and sometimes even short-, term goals. Consequently, they often fail to reach any of the goals that they set themselves.

#### Anankastia

Individuals high on Anankastia have a very clear and detailed personal sense of perfection and imperfection that extends beyond the typical standards of their community. They believe strongly that everyone should follow all rules exactly and meet all obligations. Like Mr. R, individuals high on Anankastia may redo the work of others because it does not meet their perfectionistic standards.

Individuals with this trait strongly believe in controlling themselves and situations to ensure that their perfectionistic standards are met. They have a preoccupation with social rules and obligations and what should be considered right and wrong. They focus intensely on detail and are highly systematic and organized to the point of being rigid. Their intensity of focus on issues or orderliness, neatness and structure frequently leads to interpersonal difficulties because they expect these same high standards from everyone else. They may also have extreme difficulty making decisions as they are not sure that they have considered every aspect of the situation.

Applying the same rules of order to their emotional and behavioural expression such that they do not express emotions or only in a very minimal way is common manifestation of the trait. Their extreme planfulness means that they are often incapable of spontaneity or of making changes to their schedule. They are very risk aware and so are highly unlikely to engage in any activity that would be likely to have a negative consequence.

#### Borderline Pattern

The original intention with the new ICD-11 classification was to end after the identification of trait domains. Extensive concern was expressed by the clinical and academic community about the changes to the classification and in particular about continued access to treatments (Herpertz et al., 2017). Following discussions with representatives from concerned groups, a concession was agreed primarily to ensure that no one was disadvantaged by the removal of the ‘borderline’ / ‘emotionally unstable’ personality disorder diagnosis. In some jurisdictions without this diagnosis payment for some specialist treatments would be unavailable and so in order to limit this possibility a borderline pattern specifier was introduced which essentially has the same diagnostic features for BPD as in DSM.

#### The Special Case of Adolescents

One noteworthy feature of the ICD-11 classification is the removal of any age specification for the diagnosis. Previously diagnosis was either forbidden in under 18s or strongly discouraged and reluctance to diagnose in clinicians was well documented (Chanen et al., 2020). The reasons for this were primarily a concern about assigning a stigmatising diagnosis to a young person especially when their personality was still in development. Whilst this concern is legitimate, it resulted in the paradoxical position that a disorder known to begin in adolescence could not be identified and addressed because of the restrictions on classification. With ICD-11, clinicians can make a diagnosis and this opens up the opportunity for early intervention for young people whose behaviours may meet the essential requirements for a diagnosis and yet because of their youth these behaviours may be less entrenched and more open to change (Chanen et al., 2020). Caution is still required, however. As discussed earlier, young people may demonstrate concerning behaviours that may be better accounted for by other diagnostic descriptions e.g. what could be described as personality disorder with traits of detachment and anankastia may be much better accounted for by an autism spectrum diagnosis or their behaviour may be a response to adverse environmental circumstances. Thorough assessment and consideration are required.

## Assessment

Given the risks and potential harms of a personality disorder diagnosis careful assessment is required. Typically, clinicians utilise clinical interviews, observation and psychometric assessment, although, the ICD-11 system is designed to be used without use of formal psychometric measures and, in some non-specialist settings, this will be all that is available. Robust assessment requires more than one meeting with the person and would also involve discussion with people who know the person well (with the consent of the person being assessed). A comprehensive clinical interview should begin with the person’s current functioning and its history paying particular attention to a developmental history, early adversity and trauma. Throughout the clinician will seek to establish the breadth of areas which are impacted, considering functioning in social, educational, occupational and familial roles. Sufficient duration of difficulties must be considered and, as discussed earlier, alternative explanations, diagnoses or contextual factors must be ruled out.

Newly developed measures are now available to measure both severity and trait domains to augment clinical interview and observations. The ICD-11 Personality Disorder Severity Scale (PDS-ICD-11; Bach et al., 2021) is a 14-item measure that shows promise and provides a rapid assessment of the severity of personality dysfunction. Bach et al. (2017) and Sellbom et al. (2020) describe a method of scoring the ICD-11 trait specifiers utilising the Personality Inventory for DSM-5. Clark et al. (2021) have recently developed a self-report measure of both self and interpersonal functioning as well as the trait domains. For clinicians interested in a more nuanced assessment of the facets that comprise the trait domains, Oltmanns and Widiger (2020) have developed a 121-item facet-level assessment of the ICD-11 model. The recently modified PID5BF+ captures both ICD-11 and DSM-5 trait domains using three facets per domain (Bach et al., 2020).

## Conclusion

ICD-11 personality disorder diagnosis moves away from a Schneiderian typology that has governed personality disorder classification for almost a century and established the connection with the psychological study of ‘normal’ personality structure. In so doing ICD-11 provides an opportunity to root our conceptualisations of a person’s established patterns of emotions, thoughts and behaviour within a psychological case formulation that understands these patterns as a person’s best attempts at functioning in often less than ideal environments. Whilst transitioning away from well-understood and familiar concepts presents a challenge, the simplified structure of the classification opens up potential benefits in terms of simplicity and clinical utility, increased awareness of risk and better matching of resource intensive therapies to severe presentations. How far these benefits are realised will depend upon clinicians embracing the new classification, on researchers further developing measures to capture the new method of classifying and on treatment developers evaluating their treatments using the new structure.
